# The impact of Juvenile Drug Treatment Courts (JDTC) implementing Federal Evidence-Based Guidelines on recidivism and substance use: multisite Randomized Controlled Trial (RCT) and Regression Discontinuity (RDD) Designs

**DOI:** 10.1186/s40352-021-00158-2

**Published:** 2021-12-06

**Authors:** Matthew L. Hiller, Steven Belenko, Michael Dennis, Barbara Estrada, Chelsey Cain, Juliette R. Mackin, Raanan Kagan, Lauren Pappacena

**Affiliations:** 1 Department of Criminal Justice, Temple University, Philadelphia, USA; 2 Chestnut Health Systems, Bloomington, IL USA; 3 NPC Research, Portland, OR USA; 4 Carnevale Associates, LLC, Washington, DC, USA

**Keywords:** Randomized controlled trial, Regression discontinuity design, Juvenile drug treatment courts, Guidelines, Recidivism, Substance use

## Abstract

**Background:**

Juvenile drug treatment courts (JDTC) have struggled to define themselves since their inception in 1995. Early courts followed a format similar to adult drug courts, but these did not address the unique needs of juveniles, which led to the creation of 16 Strategies by a consensus panel of practitioners and researchers. But, like the first JDTCs, research with courts following these strategies failed to provide convincing evidence that this “model” was associated with significant reductions in recidivism or drug use. More recently, a new set of evidence-based guidelines were developed through meta-analyses commissioned by the Office of Juvenile Justice and Delinquency Prevention (OJJDP, 2016).

**Method:**

OJJDP provided funding for a rigorous multi-site evaluation of the guidelines. This study protocol paper for the Juvenile Drug Treatment Court (JDTC) Guidelines Cross-Site Evaluation presents research designs for the comparison of youth outcomes from 10 JDTCs compared with 10 Traditional Juvenile Courts (TJCs) in the same jurisdictions. Two sites opted into a randomized controlled trial (RCT) and eight sites chose to follow a regression discontinuity design (RDD). Youth data are captured at baseline, and at 6- and 12-month follow-ups using an interview comprised of several standardized assessments. The youths’ official records also are abstracted for recidivism and substance use information. The degree to which the evidence-based guidelines are implemented at each site is assessed via an in-depth court self-assessment collected at baseline and again 2 years later and via structured site visits conducted once during implementation.

**Discussion:**

As a field-based trial, using both RCT and RDD designs, findings will provide important, policy-relevant information regarding the implementation of the OJJDP evidence-based guidelines, including the degree to which JDTCs adopted and/or modified these practices, their relative impact on recidivism and substance use, as well as the degree to which JDTCs differ from TJCs. Specific inferences may be drawn about whether following or not following specific guidelines differentially impact youth outcomes, yielding recommendations about the translation of this information from research-to-practice for potentiating the broader adoption of these guidelines by JDTCs nationwide.

**Clinical trials registration:**

This was not an NIH supported trial. The funder, OJJDP/NIJ, instead required publishing the design with even more information at https://www.ojp.gov/ncjrs/virtual-library/abstracts/juvenile-drug-treatment-court-jdtc-guidelines-cross-site-evaluation.

## Background and rationale

### Evolution of juvenile drug court standards

To address the significant increase in youth adjudicated for substance use and related offenses, juvenile drug treatment courts (JDTC) were first established in 1995, emulating the first adult drug treatment court model established in 1989, in Miami, Florida. Unlike adult drug courts that received extensive attention by researchers, practitioners, and policy makers, JDTCs flew “under-the-radar” for many years with few effectiveness studies conducted, with most of these studies showing a negligible and mixed impact on recidivism and drug use (Dennis et al., 2016; Ives et al., 2010; Wilson et al., 2019).

From their beginning, JDTC practitioners and researchers noted that the key components of the adult drug treatment court model (National Association of Drug Court Professionals (NADCP), 1997), lacked important elements needed to address the specific, unique needs of youth in the juvenile justice system (Dennis et al., 2016; Hiller et al., 2010; Stein et al., 2015). For example, absent from the 10 Key Components was a specific focus on families. Therefore, revisions were needed to indicate strategies designed to engage the family, like family therapy, to increase chances for improved outcomes for the youthful offenders.

Building on the experiences of the first decade of JDTCs, a workgroup was formed to develop youth-focused guidelines, resulting in the publication, *Juvenile Drug Courts: Strategies in Practice* (Bureau of Justice Assistance (BJA), 2003). Often referred to as the 16 Strategies, they retained key components, including an interdisciplinary non-adversarial team, involvement of a judge, and on-going evaluation and planning, and modified several others. For example, Strategy #8 called for tailoring treatment to the developmental needs of adolescents. Strategy #12 focused on recognizing and engaging the family as a valued component (often interpreted as a suggestion to use family therapy). Strategy #11 focused on the strengths of youth and their families, and Strategies #9 and #10 called for providing gender appropriate- and culturally-sensitive treatment, respectively. These strategies were adopted by the National Council of Juvenile and Family Court Judges (NCJFCJ) as the training curricula for jurisdictions planning new JDTCs.

A multi-site process evaluation by Butts and Roman (2004) showed significant variation in how JDTCs were implemented, consistent with other studies (e.g., Hiller et al., 2010; Mericle et al., 2014; Sullivan & Latessa, 2011). This finding underscored the difficulty in determining the fidelity of implementation according to the Strategies, prompting van Wormer (2010) to develop a survey designed to measure the degree to which courts were following the 16 Strategies. Concurrently, a growing effectiveness literature, summarized in several meta-analyses (e.g., Mitchell et al., 2012; Tanner-Smith et al., 2016a, 2016b; Wilson et al., 2019) continued to show an inconsistent impact of JDTCs, and JDTCs seemed to become imperiled, with one state closing all of its JDTCs. To bolster the JDTC model, a demonstration program merged the evidence-based practice, *Reclaiming Futures*, with the juvenile drug court (Dennis et al., 2016).

### Juvenile drug treatment court guidelines

By 2014, with continued concern over the mixed findings regarding the impact of JDTCs, the Office of Juvenile Justice and Delinquency Prevention (OJJDP) launched a 6-year plan to better understand the current state of research on JDTCs, develop a new set of guidelines based on this evidence, and evaluate the effectiveness of the new guidelines.

**Fig. 1:**
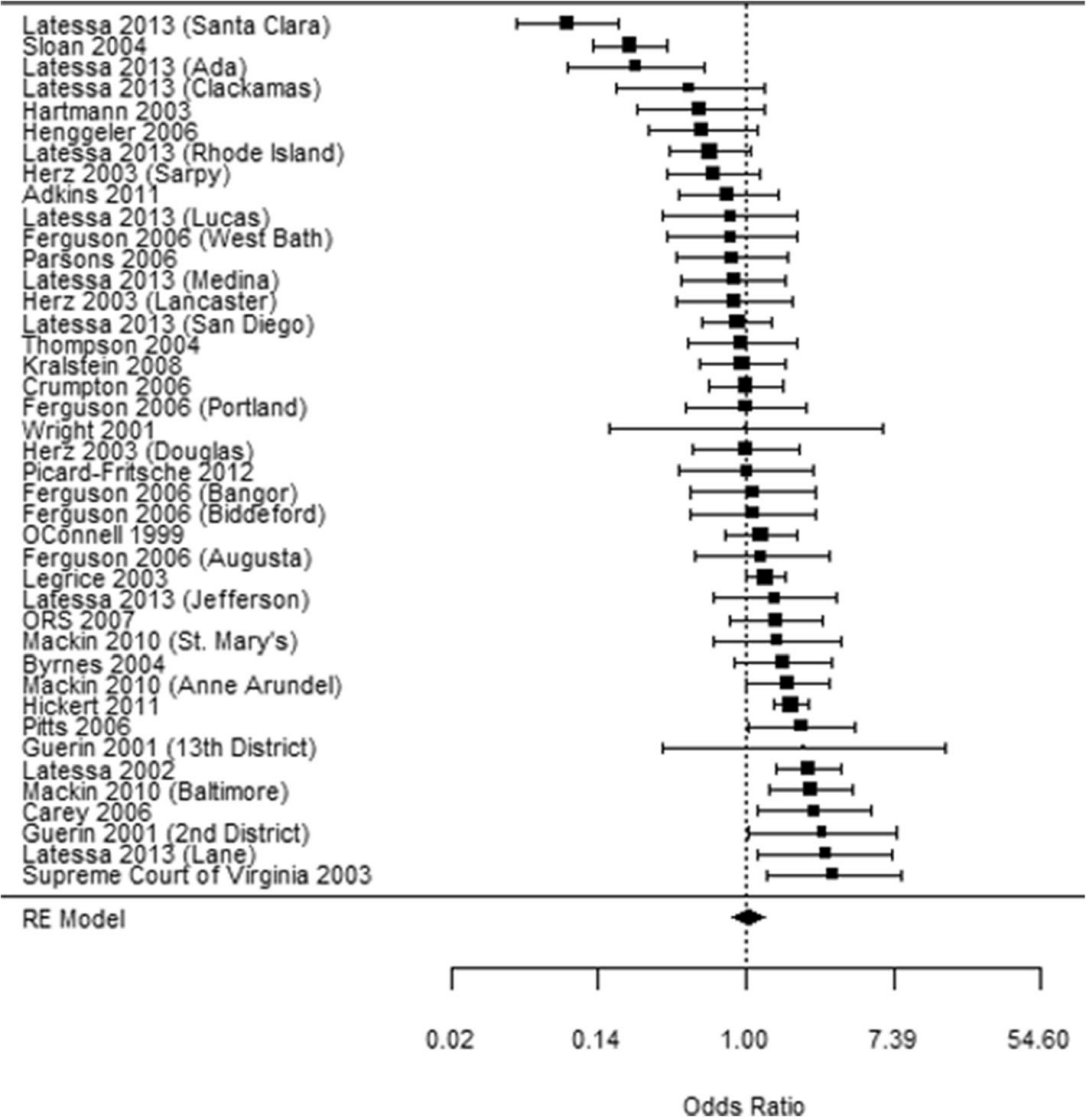
Forest Plot of Effect Sizes from Tanner-Smith et al. (2016a)

The first phase of the OJJDP effort involved conducting a meta-analysis of studies that included a comparison of JDTCs to Traditional Juvenile Courts (TJCs), as well as convening panel discussions with expert practitioners and researchers. The meta-analysis examined 41 experimental or quasi-experimental evaluations and found, on average, no differential effect on general recidivism, recidivism for drug law violations, and drug use (Tanner-Smith et al., 2016b). However, significant variation in the effect sizes was observed (Fig. 1). For example, the three studies at the top of Fig. 1 [i.e., Latessa et al., 2013, (Santa Clara); Sloan III et al., 2004; and Latessa et al., 2013 (Ada)] found that the JDTC did worse than TJC. The 29 studies in the middle [i.e., Latessa et al., 2013, (Clackamas) through Byrnes & Hickert, 2004] found no clear differences (i.e., the 95% confidence intervals (CI) for the effect sizes included the odds ratio value 1.0), and eight of the nine studies at the bottom of the figure [i.e., Mackin et al., 2010 (Anne Arundel), through Supreme Court of Virginia and Virginia Department of Criminal Justice Services, 2003] found that the JDTC did better than TJC (i.e., the 95% CI did not include an odds ratio of 1.0). Some other key findings of the review included: a) the results were similar for recidivism overall and for drug-related crime; b) JDTCs often were not always focused on serving those youth who might benefit the most from them; c) substance use treatment initiation and engagement were often problematic; and d) youth were often referred to psycho-educational substance use education and treatment that were not evidence-based practices. These latter findings suggest a possible reason (i.e., implementation failure) why some JDTCs have better outcomes than others (Sullivan & Latessa, 2011). Perhaps outcomes would have been different had JDTCs been implemented to include evidence-based practices and/or more closely adhere to a set of research-based guidelines.

**Fig. 2:**
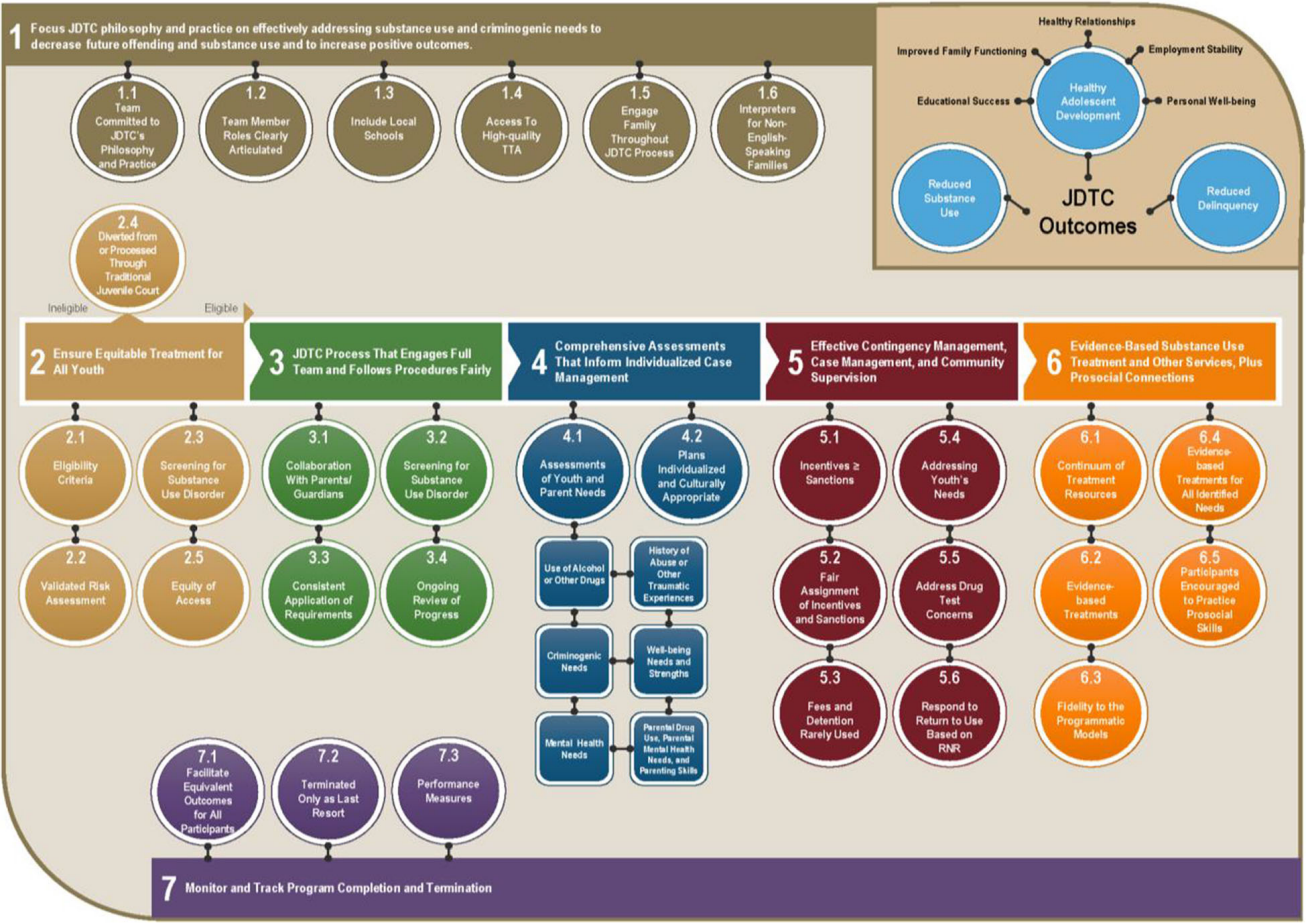
OJJDP Evidence-Based Guidelines for Juvenile Drug Treatment Courts (JDTCs). This figure is from the Office of Juvenile Justice and Delinquency Prevention (OJJDP, 2016) *Juvenile Drug Treatment Court Guidelines*. Washington, DC: U.S. Department of Justice, Office of Justice Programs. NCJ 250368. https://ojjdp.ojp.gov/programs/juvenile-drug-treatment-court-guidelines

Therefore, the Office of Juvenile Justice and Delinquency Prevention developed and published the evidence-based *Juvenile Drug Treatment Court Guidelines* (Office of Juvenile Justice and Delinquency Prevention (OJJDP), 2016) to help JDTCs implement more effective practices reduce the use of ineffective ones, as well as to be consistent with other juvenile justice reform efforts. The JDTC Guidelines (Fig. 2) are organized into 7.

## Objectives

1. Focus JDTC philosophy and practice on effectively addressing substance use and criminogenic needs to decrease future offending and substance use and increase positive outcomes;
2. Ensure equitable treatment for all youth by adhering to eligibility criteria and conducting an initial screening;
3. Provide a JDTC process that engages full team and follows procedures fairly;
4. Conduct comprehensive needs assessments that inform individualized case management;
5. Implement contingency management, case management, and community supervision strategies effectively;
6. Refer participants to evidence-based substance use treatment, to other services, and for prosocial connections;
7. Monitor and track program completion and termination.

Within each area, these Objectives were further operationalized into 2 to 6 specific Guidelines (31 total). The current study was developed to test the implementation of these Guidelines and their impact on JDTC outcomes relative to TJC. The following section describes the research design of the study, including its goals, research methodology, and key considerations, particularly implementation fidelity, essential to the interpretability of study findings.

### Evaluation goals and questions

This study is the first cross-site evaluation of the JDTC Guidelines (Office of Juvenile Justice and Delinquency Prevention (OJJDP), 2016). As presented in Table 1, along with specific measurement strategies, this study has 4 major goals, including (1) Determine the extent to which it is feasible to implement the 2016 JDTC Guidelines and the kinds of adaptations courts make to use them; (2) Examine the impact on youth of the JDTC relative to TJC; (3) Identify whether there is evidence for some of the Guidelines being more or less important and/or not important; and (4) Recommend changes to the Guidelines. The specific research questions are:

1. Do youth with substance use disorders (SUD) experience more positive outcomes if assigned to a JDTC vs. TJC?
2. Are different interpretations of the Guidelines by the courts associated with better outcomes?
3. Are there certain guidelines that, if present, are associated with better outcomes?
4. Are there guidelines that, if absent, do not seem to be associated with worse outcomes?
5. Do some of the seven broad Objectives of the guidelines have a stronger association with outcomes than others?
6. Is there evidence that instances of NOT following the guidelines produce worse outcomes?

**Table 1:** Evaluation Goals and Questions and Corresponding Measurement Strategy

Goals	Baseline Interview	Follow-Up Interviews	Records Search	Court Self-Assessment	Site Visit Protocol
Determine the extent to which it is feasible to implement the 2016 JDTC Guidelines and the kinds of adaptations courts make to use them				X	X
Examine the impact on youth of the JDTC relative to TJC	X	X	X		
Identify if there is evidence for some components of the Guidelines being more or less important or not important	X	X	X	X	X
Recommend changes to the Guidelines based on above	X	X	X	X	X

## Method

### Research design and court sites

**Fig. 3:**
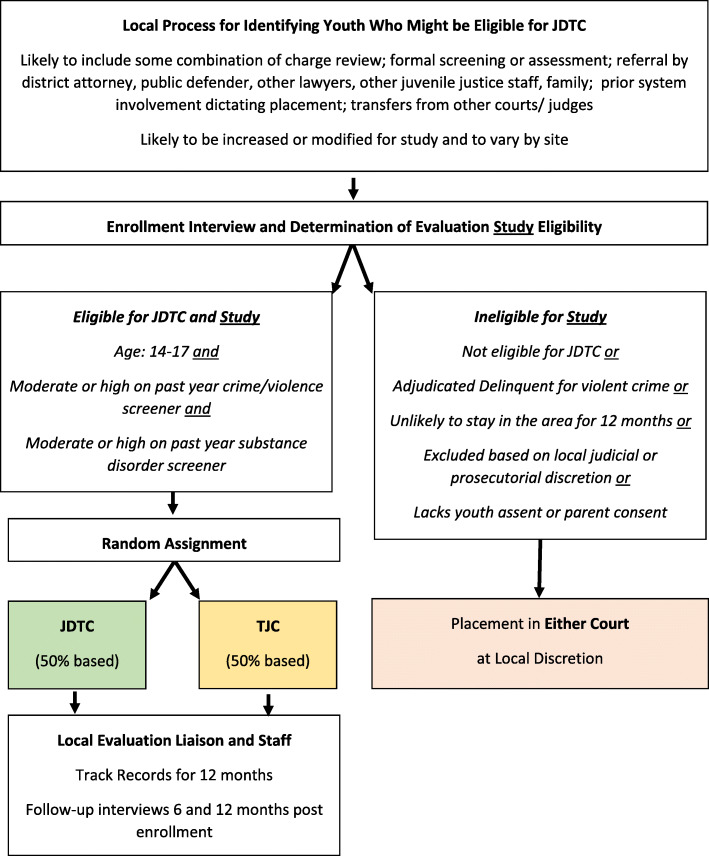
Procedure for Recruitment and Random Assignment of Youth to Study Group (JDTC or Traditional Juvenile Court (TJC) for the Multisite Randomized Controlled Trial (RCT)

The cross-site evaluation design involves two parallel studies across 10 sites (defined as a county jurisdiction with a participating JDTC and a participating TJC). As shown in Fig. 3, in two sites, youth who are eligible for JDTC ***and*** TJC are randomly assigned to JDTC vs. TJC. This design provides the most rigorous and direct test of JDTC’s impact relative to TJC. This RCT will be reported according to CONSORT guidelines (Grant et al., 2018).

**Fig. 4:**
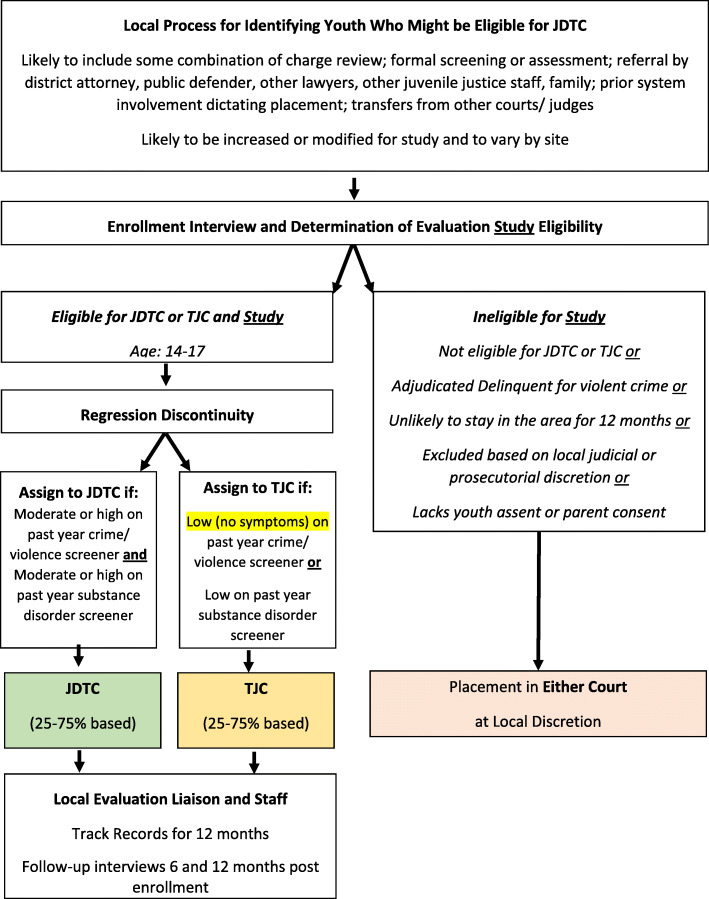
Procedure for Recruitment and Assignment of Youth to Study Group (JDTC or Traditional Juvenile Court) for the Multisite Regression Discontinuity Design Study

As shown in Fig. 4, in the remaining eight sites, youth who are eligible for JDTC ***or*** TJC are assigned to the most appropriate court using a regression discontinuity (RD) algorithm. Per the guidelines, this design means that youth who are at moderate to high risk of recidivism ***and*** have a SUD will be assigned to JDTC and the rest to TJC. Here, the impact of JDTC will be estimated relative to the expected outcome (recidivism) using regression and the TJC data. This design will also provide a test of the Guidelines’ recommended eligibility criteria.

### Research participants

As shown in Figs. 3 & 4, to be eligible for the cross-site evaluation study, youth must be age 14 to 17 and involved in a juvenile court with a judge presiding (i.e., not including diversion or informal supervision without a judge). For RCT, they must also be eligible for JDTC and TJC. For RDD, they must be eligible for JDTC or TJC. For logistical and practical reasons, youth are excluded from the evaluation study: (a) if they have been adjudicated guilty of a violent offense; (b) if they are expected to move out of the jurisdiction within 12 months; (c) based on judicial or prosecutorial discretion prior to assignment (e.g., someone also being charged with sex crimes or violent offense but not yet convicted); and (d) when the JDTC is already at full capacity. Participation is voluntary and requires the informed assent of the youth and the informed consent of the parent/guardian. After the research team assigns the youth to a condition, the courts can override the assignment and place the youth elsewhere and consent can be withdrawn. Target recruitment numbers were 150 for each of the 10 study sites, yielding a total of 1500 youth with a minimum of 500 in JDTC and a minimum of 500 in TJC. The numbers are expected to vary somewhat as the RD design is based on presenting need/risk (not a fixed ratio).

### JDTC standard training and technical assistance

Two training and technical assistance (TTA) providers were funded by OJJDP to develop training materials and help courts implement the JDTC Guidelines. American University (AU) and the National Association of Drug Court Professionals (NADCP) work with three JDTC grantees who received independent grants for 5 years, and the National Council of Juvenile and Family Court Judges (NCJFCJ) work with seven learning collaborative courts that received training and support for over 3 years. These courts have been approved by NCJFCJ to serve as model JDTCs that are interested in changing practices to improve operations and outcomes. Monthly calls between the research team and the TTA providers and sites are used to monitor training, and monthly calls with research sites help the research team stay abreast of site-level data collection activities and answer questions about the research design.

### Data collection procedures

As show in Table 1, data collection is guided by the goals of the study. It involves measures at both the study participant level (i.e., baseline and follow-up interviews, administrative court records) and the site level (i.e., Court Self-Assessment and research site visits). The former measures are specifically focused on comparison of JDTC and TJC interactions with youth (supervision, drug testing, service provision, etc.), which are hypothesized to determine efficacy of JTDC, and the latter measures are focused on understanding the feasibility and degree of implementation of the guidelines and determining whether some have a greater impact than others. Data collection is facilitated by site-based research liaisons (a person local to the research site who is trained on the study procedures and who serves as the first-tier responder to questions from program and evaluation staff), by facilitating communication between the site and evaluation teams to obtain the youth survey data, as well as the abstraction of administrative data from the youths’ juvenile justice records including local and statewide jurisdictions. The data on the implementation of the courts are collected via semi-structured multiple-day site visits by researchers at Carnevale Associates, LLC, and the collection of the Court Self-Assessment data is coordinated by researchers at NPC Research.

#### Youth survey

As show in Table 2, a baseline survey is collected from youth prior to their assignment to study condition, and again at 6 and 12 months following (i.e., follow-up) their assignment. Youth receive an incentive of $5 for baseline, $15 for 6-month follow-up, and $20 for 12-month follow-up (for a maximum total of $40) disbursed on McDonald’s gift cards. The baseline and follow-up surveys are designed to take 25 to 30 min to complete, and include numerous empirically validated assessments, including the Global Assessment of Individual Need Q3 (GAIN Q3; Titus et al., 2013), Mental Health Continuum Short Form measure of mental well-being (MHC-SF; Keyes & Simoes, 2012; McGaffin et al., 2015), the Family Effectiveness Measure (FAM; McCreary et al., 2013), the National Mentor Resource Center’s (NMRC) “Very Important non-parent Adult” (VIA; Herrera et al., 2007), Social Environment Scale (SES; Godley et al., 2005), the structured activity scale from the National Mentoring Resource Center Out of School Time (OST; Scales et al., 2006), and the Global Assessment of Individual Need Short Screener (GAIN-SS; Dennis et al., 2006). An evaluation liaison and up to two additional staff in some sites have been trained and certified in administering the study survey by the cross-site evaluation team led by Chestnut Health Systems. These staff administer the interview on the project’s web-based software, GAIN ABS, in person or by phone/Zoom® video conferencing to youth after obtaining informed assent/consent from the youth and their parent/guardian (baseline), as well as at 6 and 12 months later. In response to a drop off in follow-up surveys after COVID emerged, the study also introduced the option for youth to self-administer the follow-up via the web service. Youth survey data are downloaded quarterly and reviewed for inconsistencies and/or missing data; data from these instruments will also be compared to the published psychometrics from their original sources.

**Table 2:** Summary of Data Collected for the JDTC Guidelines Cross-Site Evaluation Project

Data Collection Instrument	Source of Instrument	Data Collection Interval	Party Coordinating Data Collection
Youth Surveys Sections		Baseline, 6 & 12 Month Follow-up	Local Liaison, Chestnut Health Systems
A. Exclusion and Consent Checklist, start time, time anchoring	GAIN Q3 (Titus et al., 2013)	”	”
B. Background Information	GAIN Q3 (Titus et al., 2013)	”	”
WB. Wellbeing	Mental Health Continuum Short Form (Keyes & Simoes, 2012; McGaffin et al., 2015)	”	”
FE. Family Environment	Family Effectiveness Measure (FEM; McCreary et al., 2013) and Very Important Adult (VIA; Herrera et al., 2007) questions from National Mentoring Resource Center (NMRC)	”	”
SP. School and Peers	GAIN Q3 (Titus et al., 2013), Social Environment Scale (SES; Godley et al., 2005) and Out-of-School Time (OST) structured activity scale from National Mentoring Resource Center (Scales et al., 2006)	”	”
RB. Risk Behavior	GAIN Q3 (Titus et al., 2013)	”	”
MH. Mental Health	GAIN Q3 (Conrad et al., 2010; Conrad et al., 2012; Titus et al., 2013)	”	”
SU. Substance Use	GAIN SS (Dennis et al., 2006; Garner et al., 2013; Riley et al., 2007)	”	”
CV. Crime and Violence	GAIN SS (Conrad et al., 2010; Dennis et al., 2006; Garner et al., 2013)	”	”
Z. End	GAIN Q3 (Titus et al., 2013)	”	”
XADM Administration	GAIN Q3 (Titus et al., 2013)	”	”
Youth Record Abstraction	JJTRIALS youth records data funded by NIDA (Belenko et al., 2017) and JDTC Guidelines funded by OJJDP (2016)	Continuously throughout project	Local Liaison, Chestnut Health Systems
JDTC Court Self-Assessment	JDTC cooperative tool funded by OJJDP to measure implementation of the 2016 JDTC Guidelines	Spring 2018, Spring 2020	NPC Research
TJC Self-Assessment	Abbreviated version (omits specific JDTC-focused) questions of JDTC Court Self-Assessment	Spring 2018, Spring 2020	NPC Research
Site Visit Protocol	RWJ RF JDTC Evaluation (Greene et al., 2014) and JDTC Guidelines funded by OJJDP	Fall 2018 to Winter 2020	Carnevale Associates, LLC

Notes: *GAIN Q3* Global Assessment of Individual Need Q3, *GAIN SS* Global Assessment of Individual Need Short Screener, *JJTRIALS* Juvenile Justice Translational Research on Interventions in Adolescents in Legal Settings, *NIDA* National Institute on Drug Abuse, *OJJDP* Office of Juvenile Justice and Delinquency Prevention, *RWJ* Robert Wood Johnson, *RF* Reclaiming Futures

#### Youth juvenile justice records

Based on a procedure used in a prior multisite study of a process improvement intervention designed to reduce unmet substance use service needs among juveniles on probation (Dennis et al., 2019, b), juvenile justice records are abstracted by JDTC staff at each local research site to record each study participant’s history of prior arrests, current charges, changes in courts, dispositions, and rearrests during the 12 months following assignment to study condition. In addition, the sites extract data on assessment and treatment using the Behavioral Health Services Cascade framework (Belenko et al., 2017; Dennis et al., 2019, b). Abstracted data are uploaded monthly and the site evaluation liaison is given feedback on the quality of site records data submissions each quarter.

#### Court self-assessment (CSA)

JDTCs and TJCs are scheduled to complete a court self-assessment in spring 2018 and again in spring 2020 to describe the degree to which their “current” practices are similar or different from the 2016 JDTC Guidelines. Usually these will be completed by the JDTC coordinator or an individual serving in the same role (for the JDTC self-assessment) or a senior official in the local juvenile justice system (for TJC self-assessment), these assessments gather information across 14 content areas, including background (e.g., JDTC inception date, memoranda of understanding across partner agencies, and stages), JDTC eligibility, referral and entry procedures, risk and need assessment, composition and training of the team/staff, family and school engagement, treatment, case planning and other services provided to participants and their families, pre-hearing meetings and court sessions, court responses (e.g., incentives, sanctions, and therapeutic responses), drug testing, program/court completion or discharge, data collection, and estimates of characteristics of participants (e.g., % who use alcohol, race/ethnicity, and % who complete or are unsuccessful in the JDTC).

Embedded within the CSA are detailed questions that directly assess variables pertinent to the seven objectives (and numerous sub-objectives) outlined within the JDTC Evidence-Based Guidelines. For example, in relation to the Objective #4 (“Conduct comprehensive needs assessments that inform individualized case management”), the CSA queries whether participants are assessed for risk of reoffending using either an established risk assessment instrument (e.g., Youth Level of Service/Case Management Inventory, YLS/CMI) or less formal risk assessments, and the level of team training on these tools. Also, courts are asked whether they use needs assessments (like the Addiction Severity Index or Global Appraisal of Individual Needs), how they are used (e.g., determine eligibility, identify youth need, and identify family needs), and whether they are used to inform the development of individualized case plans for JDTC participants or other court-involved youth (TJC). Numerous possible case plan elements are queried, including type of case management and services (e.g., office-based outpatient group treatment, home-based family treatment, mental health counseling, parenting classes, and gender-specific services). The CSA also gathers information on whether the court has incorporated evidence-based interventions to address specific needs, such as Motivational Enhancement Therapy with Cognitive Therapy (MET/CBT), Functional Family Therapy (FFT), and contingency management (CM).

In addition to a gaining a deep understanding of how the court operates, the repeated administration of the CSA will enable comparisons between the baseline and follow-up CSAs to determine what changes occurred in site operations and guideline adherence over time. This comparison is important because it will provide information on whether courts adopted additional practices consistent with the JDTC Guidelines, or dropped practices they found difficult to implement and sustain.

#### Traditional juvenile court self-assessment (TJCSA)

Because assignment to condition will involve youth receiving services-as-usual via the TJC within the same jurisdictions as the JDTCs, it was imperative that the TJCs be assessed as well because many include some of the same components as JDTC, and several are a part of concurrent juvenile justice reform efforts. TJCs and JDTCs also use some of the same staff and treatment resources and staff are likely to change over time. The TJCSA was an abbreviated version of the CSA, with the sections pertinent only to JDTCs omitted.

#### Site visits

Data on the operations of JDTC and (to a lesser extent) TJC at each research site also are collected by a multi-day site visit conducted once during the study period and attended by two researchers. Site visit data are collected using two distinct measures: 1) semi-structured interviews conducted with JDTC staff and 2) observation of staffing and court operations. The visits aim to examine local adjudication processes, how the JDTC Guidelines are implemented and interpreted within local context, other specialized dockets/services (such as diversion outside the JDTC), and any unique or complex features not necessarily obtained using the CSA. Special attention is paid to understanding potential overlap between JDTC and TJC in terms of judges, community supervision staff, and substance use treatment program access, and to collecting data that may confirm or augment CSA findings. Extensive field notes are collected during each site visit, from which a lengthy report summarizing how each site operates is created. A logic model is also developed to depict how each program enrolls individuals to treatment. Logic models are developed and confirmed with each site to correct any omissions or inaccuracies.

### Primary outcomes

#### Recidivism

For this study, recidivism is measured both by self-report on the follow-up surveys, as well as through juvenile justice records abstraction which includes information retrieved from local and statewide databases on new arrests. Consistent with the OJJDP’s recidivism workgroup (Harris et al., 2011), operational definitions for recidivism will be derived separately for self-report and official records, as well as a combination of these information sources, and will focus on new arrests following assignment to study condition, with measures reflecting whether any new arrest occurred (0 = No, 1 = Yes), the number of new arrests, as well as subsets of these arrests based on major offense types including property, violent, public disorder, and substance possession (including alcohol). Specific arrest dates will be collected for each, enabling analyses to consider the latency (i.e., length of time) between study assignment and date of arrest.

#### Substance use

Substance use problems are measured using the GAIN-SS’s Substance Disorder Screener (SDScr; Dennis et al., 2006) and the number of days of substance use, both components of the youth surveys. The SDScr has five items measuring recency of symptoms related to weekly use; time spent consuming drugs; drug-related problems; reduced involvement at work, home, or school; and withdrawal. It has been recommended by the National Institutes of Health (NIH; https://www.phenxtoolkit.org/protocols/view/560102?origin =search) and Substance Abuse and Mental Health Administration (Substance Abuse and Mental Health Services Administration (SAMHSA), 2012) as one of the most reliable, valid, and efficient substance use screeners, and has been shown to be accurate for diagnosis (AUC = .9; Dennis et al., 2006) and sensitive to change, and to predict change in standardized educational test scores (Rattermann, 2014). The operational definition is percent days of use, which is calculated as the days of use in the prior 90 days divided by 90 minus any days in a controlled environment, and trimmed to stay between 0 to 100%.

### Secondary outcomes

The study also has multiple secondary outcomes, including measures of changes in internalizing and externalizing mental health symptoms, well-being (happiness, connectivity, self-worth), relationships with parents/guardians and other very important adults, peer risk and support, involvement in prosocial structure activities, and academic performance. Further information on these other measures is available in the detailed study design protocol prepared for OJJDP (Dennis et al., 2019, b).

## Discussion

Juvenile drug treatment courts (JDTC) have struggled to define themselves since their inception in 1993. Early courts followed a format similar to adult drug courts, but these clearly did not address the unique needs of juveniles, including developmentally-appropriate treatment services and the importance of working within the family environment. Developed by a consensus panel of practitioners and researchers from these earlier courts, a set of guidelines emerged detailing 16 strategies for JDTCs (Bureau of Justice Assistance (BJA), 2003). But, like the early JDTCs, research with courts following these strategies failed to provide convincing evidence that this “model” was associated with significant reductions in general recidivism or in drug use. Several meta-analyses found that JDTC impacts were inconsistent and inconclusive on general recidivism, recidivism for drug law violations, and drug use (Mitchell et al., 2012; Tanner-Smith et al., 2016a, 2016b; Wilson et al., 2019). Hybrid models of JDTCs were developed, with the most common of these hybrids being a type that incorporated the evidence-based practice, *Reclaiming Futures* (Korchmaros et al., 2015). The most recent focus has been on a new set of evidence-based guidelines for JDTCs, developed through meta-analyses commissioned by Office of Juvenile Justice and Delinquency Prevention (OJJDP), 2016. OJJDP also provided funding for a rigorous multi-site evaluation of these guidelines. This current paper presented the protocol for the Juvenile Drug Treatment Court (JDTC) Guidelines Cross-Site Evaluation project, including an in-depth description of the research design, research sites and samples, measures, and data collection protocol designed to complement the study goals and research questions.

The study is well-positioned to complete each goal and to answer each research question, by using multiple data sources and collection methods for both the program and youth levels. For example, several study goals and research questions are specifically focused on the feasibility of implementing the new guidelines and understanding whether different interpretations of the guidelines by the JDTCs are associated with better outcomes, or whether the presence or absence of specific guidelines lead to better/poorer outcomes. Therefore, it is particularly important for the study to use reliable, comprehensive measures of program implementation. The protocol uses a Court Self-Assessment survey that carefully measures the courts’ perception of whether and to what extent each of the specific guidelines were implemented, as well as a multi-day site visit during which researchers observe court operations and interview key stakeholders. The Court Self-Assessment is conducted twice, at baseline and again 2 years later. The timing of the Court Self-Assessment enables determination of whether programs change over time in their use and modification of the guidelines. It is important to know which guidelines are adopted and which are not sustained over the course of the study.

The multi-day site visit, which occur between the first and second administration of the Court Self-Assessment, provide a limited validity check of self- and researcher-ratings of JDTC implementation of specific guidelines. The site visits also allow researchers to ask questions related to assessment findings and obtain a level of detail about site-specific context and guideline interpretation not achievable via survey. The site visit may be particularly helpful for understanding changes in how the guidelines are or are not implemented because researchers are able to question the programs about the specific contexts surrounding these decisions.

Another significant benefit of the current study is that the self-assessment (absent JDTC-specific questions) is also completed for the traditional juvenile court (TJC) at each site. An unanswered question in the current literature is to what extent practices differ between co-located JDTCs and TJCs. It is unclear to what extent treatment intensity, court review, family therapy, mental health services, and drug testing will vary between JDTC and TJC. The greater the similarity between JDTC and TJC, the greater the likelihood that the evaluation will show null effects related to the outcome measures, similar to the Rio Hondo DUI Court evaluation, which found at the conclusion of a randomized controlled trial that it was not effective compared to the standard sanctions for DUI (Eibner et al., 2006; MacDonald et al., 2007). However, when looking for possible reasons for the null results, these researchers found a high degree of similarity, including treatment intensity, between the DUI court and the standard interventions, with the primary difference being the number of times offenders’ progress was reviewed by a judge. DUI court participants had a modestly higher number of contacts with the judge (Eibner et al., 2006; MacDonald et al., 2007). Knowing what differences exist in service availability and usage between JDTC and TJC sites, therefore, is paramount to avoiding erroneous null conclusions. Site visit interviews further augment CSA findings in this area.

Youth-level data collection procedures also evidence significant strengths. For example, self-reported data, much of which is typically not the part of official administrative records, will be used to collect both the primary (i.e., recidivism and drug use) and secondary outcomes (e.g., mental health and wellness), enabling direct comparisons between JDTC and TJC youth on each of these outcomes. Importantly, these self-reported data are collected using an interview that incorporates numerous instruments with known measurement properties for the same populations being studied. This approach significantly enhances confidence regarding data validity and reliability. Moreover, the interview data are collected three times during the course of the study; baseline, 6 months, and 12 months post-program entry. The use of multiple comparable time frames for these data enables comparison between JDTC and TJC youth on both primary and secondary outcomes at each time point, as well as change over time. When combined with the data on guideline implementation and the degree to which services overlap between JDTC and TJC conditions, analyses will be able to provide a clearer picture regarding the degree to which youth in each condition change (for better or for worse) while controlling for other potentially confounding variables.

Outcome data also are being abstracted from the administrative records, which include local and state-wide information for both JDTC and TJC youth. These data are valuable for comparison to the self-report data for establishing its concurrent validity. In addition, the degree to which findings observed in the self-reported data (i.e., between-group differences in recidivism and drug use) also are found in the administrative data will enhance the construct validity of research findings regarding the effectiveness of the JDTC programs. An added benefit of the administrative data is that specific event dates (e.g., date of a new arrest, date of a positive drug assay) will be recorded. This detail enables event history analysis of the relative risk ratios over time between groups and considers the unique aspects of data (e.g., censoring), as well as differences in risk ratios to specific time points (operationalizable in both days, weeks, and months) tested, such as time to first arrest, or number of days of negative drug tests.

Strong inferences regarding the effectiveness of JDTC vs TJC are made possible by the unique hybrid design used by this study. Both randomized controlled trials (RCT, two sites) and regression discontinuity designs (RDD, eight sites) are strong research designs for establishing the internal validity of the study. Equivalent JDTC and TJC groups in the RCT, for example, eliminate concerns related to selection artifacts related to pre-existing differences in the groups being studied. Similarly, whether mortality is a threat to the internal validity of the study involving differential dropout of youth between conditions (which results in non-equivalent groups by the end of the study) can and will be examined. The presence of an RDD has the added benefit of being more acceptable to judges and practitioners who may be uncomfortable with random assignment of youth to different programs/services. The RDD uses a pre-established cut-score involving both criminal and substance use histories to assign youth either to JDTC or TJC. Comparison of the expected outcomes for JDTC to the observed outcomes relative to those for the TJCs will indicate whether observed outcomes for the JDTCs more closely resemble those predicted for them or the outcomes following TJCs.

Gaining important insight needed for guiding policy and practice surrounding JDTC is a significant expectation of the current study. For example, this is one of only a handful of RCTs of JDTCs. As noted earlier, the question of the effectiveness of JDTCs remains unclear. If findings from our RCT show significant reductions in recidivism and drug use and improvement in secondary outcomes like mental health, it will provide additional support for the JDTC, complementing the RCTs conducted by Henggeler et al. (2006, 2012). This support would come at a critical time in the development of JDTCs, which have seen a 31% decrease in the number of operational programs between 2013 and 2020; that is, a decrease from 447 JDTCs in 2013 (Marlowe et al., 2016) to 308 in 2020 (National Drug Court Resource Center, 2021). The data collected from the Court Self-Assessment and the TJC Self-Assessment will provide important information about whether/how JDTCs differ from “standard of care” (i.e., TJC) when we analyze the JDTC and TJC pairs. It is important to determine whether JDTCs and TJCs represent largely overlapping models of services referral and delivery. If there is substantial overlap, if there are null findings between JDTC and TJC for reductions in general recidivism and drug use, this would seem to indicate that these largely overlapping models lead to similar outcomes. Therefore, local jurisdictions could decide between alternatives, adopting the approach that best fits with their local juvenile justice system. A deeper analysis of the variation in the implementation of guidelines, also facilitated through the CSA and TJCSA, as well as the site visits, represents, perhaps, the most substantial opportunity to draw possible implications regarding how JDTCs should be implemented to maximize youth outcomes. Practices associated with positive outcomes would provide actionable information to the field so that training and technical assistance providers, and funders, could then share information with programs and work to increase the use of the effective practices. However, because a single study alone is insufficient for conclusions, such findings also would necessitate the need for more controlled studies of specific guidelines (i.e., those found in our study to be correlated with better outcomes). Finally, this study presents a clear opportunity to revise the Evidence-based Guidelines based on the comparisons of JDTCs and TJCs and comparison of JDTCs that do/not implement a specific guideline. For example, the results can indicate which practices are more or less effective (so maybe the guidelines might be revised to emphasize some practices over others), as well as which practices are less likely to be implemented – where revisions could help possibly help provide additional guidance about how to implement the practices and/or potentially identify which practices are not feasible or practical to implement.

Limitations to the current study are related to the veracity of youth during interviews with research staff, the use of a non-random sample of JDTCs in the study, and the possibility of treatment cross-over effects between study conditions. With respect to youth self-reported data, interviews are collected by research staff trained in establishing rapport with the youth, and it is likely that youth are more comfortable with sharing information with research staff because they were told that their data are strictly confidential. The non-random sample of JDTCs presents the possibility that the sites chosen are not representative of JDTCs nationwide. JDTCs were recruited because they were sufficiently large enough to help ensure a sizable number of JDTC and TJC youth could be recruited for the study. Typically, juvenile treatment courts are quite small with fewer than 25 youth in the program. To gain insight into whether the sampled programs varied along other dimensions from what is seen nationally, survey data are available from a nationally representative sample of juvenile community supervision agencies, with a sizable number of these jurisdictions also implementing JDTCs nearly contemporaneously with the current study (Robertson et al., 2019; Scott et al., 2019). Comparing our research sites with these can provide insight into whether and how these programs differed from a larger national sample of JDTCs, giving some understanding, perhaps, into the generalizability of findings from this study. However, any examination in variations in guideline adherence in association with outcomes remains limited by the small number (*N* = 10 JDTC; 10 TJC) of programs examined. These analyses, therefore, will be discussed relative to future directions for research. For example, if implementation of a specific guideline is correlated with better outcomes, we will acknowledge this result as an important finding limited by the number of programs in the study that should be examined more precisely in, perhaps a RCT of that specific guideline among a multi-site sample of programs. Such a trial would give an unambiguous conclusion regarding the efficacy of that guideline in courts randomly assigned to adopt that guideline (or not). Finally, although some JDTCs in the current sample likely share some staff with their corresponding TJCs, it is explained and periodically reinforced with JDTC staff that if they also work with youth in the TJC condition that for the study to have a clearer basis for making conclusions, that JDTC-specific interventions should not “leak” into their work with the youth in the control condition. However, given this was applied research in real-world settings, it is likely that staff who thought JDTC practices may be useful chose to use them with the TJC youth as well. Regardless, the data that are collected from the Court Self-Assessment and the Traditional Juvenile Court Self-Assessment will give keen insight into the extent to which JDTCs and TJCs differed in their approaches and services delivered. Therefore, we will be able to see the extent to which apples-to-apples or apples-to-oranges comparisons are being made between study conditions at each study site.

In conclusion, strengths associated with the data collection tools and research designs have equipped the current study, the Juvenile Drug Treatment Court (JDTC) Guidelines Cross-Site Evaluation project, to address each of its goals and answer all research questions. The use of both program- and youth-level data permit the assessment of variations in guideline adherence, enabling testing of these practices in relation to youth outcomes. Having multiple types of implementation data strengthens confidence in the degree to which programs and researchers rate adherence to these guidelines. Comparison of JDTC and TJC youth from all sites enables the testing of variation in guideline adherence. The study will also analyze whether differences or similarities in service intensity impacts youth outcomes. Strong inferences regarding differences in youth outcomes are permitted by the rigorous designs used. The combined rigor of these designs will permit analysis of internal validity, construct validity, and reliability of measures and findings, as well as the extent to which guidelines were implemented by JDTCs, and whether there is considerable overlap in the services received by JDTC and TJC youth. As the first rigorous test of the OJJDP Guidelines, findings will yield important and timely feedback to the field as it considers adopting or modifying training and technical assistance for JDTCs, and findings will yield suggestions for how best to translate science into practice.

## Data Availability

Data collection instruments are available upon reasonable requests made to the corresponding author. Because the study is still underway, data are not available by request. OJJDP’s data availability policies will be followed for publishing study data sets.
